# On the Uses of Large Doses of Calomel in Diarrhœa and Dysentery

**Published:** 1867-06

**Authors:** A. D. Cosby

**Affiliations:** Calhoun, Ky.


### ARTICLE II.

### *On the use of Large Doses of Calomel in Diarrhœa and Dysentery.* By A. D. Cosby, M. D., of Calhoun, Ky.

I have practiced for many years upon the supposition that the effects of a large dose of calomel are very different from those resulting from a small one, or from a small one frequently repeated, until the quantity given is equivalent to that of a large one. While I do not claim the honor of originating the idea here suggested, I often meet with practitioners who are ignorent of the fact, or are not disposed to acknowledge its importance. I hope I will not be trespassing upon the indulgence of the readers of the Journal, by pressing its claims upon their consideration, with such facts as I have observed, and such arguments as I may be able to adduce. By a large dose of calomel, I mean *twenty*, and by a small dose, *five* grains.

Calomel is seldom given as a purgative, for the reason that it is slow and uncertain in its actions, and hence, much more unreliable than many other articles belonging to the class of cathartics. But when the secretions of the biliary organs are out of order, it is given for their rectification, either alone, or in combination with some purgative, for the purpose of increasing its activity. Connected with disorders of these organs, there is generally an irritable condition of the stomach and alimentary tube, when I have always found that a scruple dose is much more effectual in moderating inordinate activity of the stomach and intestinal canal, in arresting the copious serous secretions of the bowels usually present under such circumstances, and in arousing the liver into action, which is apt to be at fault, than any smaller quantity. While five or ten grains of calomel will usually increase the peristaltic action of the bowels, and cause the evacuations by irritating the stomach and bowels, and thus cause the mucous and serous secretions to be frequent, liquid, and exhausting, twenty grains will commonly bring down two or three consistent and bilious stools. Calomel, either alone or in combination, is mostly depended on to treat cases of irritable bowels, in which watery evacuations are passed, and hence it is important to know what quantity is calculated to do most good.

I have long since made up my mind on this point, and constantly keep in view the marked difference in the effects that result from large and small doses of calomel. I am perfectly satisfied that the efficacy of this remedy mainly depends, in bowel diseases, upon the fullness of the dose given, and hence, am prepared to account for the very contradictory effects it has produced in the hands of those who gave it only in small doses. That they should have but little confidence in it, is in no way strange to me, for they give it, as I humbly think, in quantities that are calculated to do more harm than good.

I deem it entirely unnecessary to enter into any theoretical explanation of the cause of the difference under consideration. Is it an incontestable therapeutical fact of importance? If so, then it will answer the purposes of the Journal, which are, as I understand, to promote practical, and discourage hypothetical medicine.

I could adduce from personal experience, hundreds of facts in conformation of the correctness of my position; but to do so, would be merely to lengthen my article without increasing its value. I will, however, introduce a few illustrative examples from the multitude that I have encountered along the pathway of a medical experience of twenty-five years, principally in order to contrast the results of small and large doses, as they have been displayed in the same cases.

In 1852, I was called to see a boy six years of age, who was laboring under an attack of diarrhœa of ten days duration. His discharges were watery and exhausting, and connected with an irritable condition of the stomach. The physician who was in charge of the case had treated it with one grain doses of calomel, in combination with Dover's powders, repeated at regular intervals. The patient had grown weaker and more emaciated from day to day, without the slightest apparent improvement in either the frequency, quantity, or nature of his evacuations. I prescribed one ten grain dose of calomel, against the protest of the attending physician.

On my return, twenty-four hours after my first visit, I learned that the patient had had but one action from the bowels during my abscence, which occurred in half hour after the calomel was given. A small dose of castor oil completed the cure. Upon what principles can the beneficial effects of the ten grain dose of calomel be explained, unless it be upon the hypothesis that a large dose of calomel acts as a sedative on the capillary vessels of the mucous coating of the stomach and intestines, while a small one irritates it?

In 1857, Mr. E——— was attacked violently with dysentery. His physician treated his case with broken doses of calomel, in connection with various astringents, for twelve days. Instead of improving, he daily grew worse and worse. The tormina and tenesmus were intensely severe. The mucous and bloody discharges were frequent and copious. His family became alarmed about his condition, and in their despair sent for me. Prescribed the following:

℞—Calomel, 15 grs.
Pulv. Opium, 1 gr.
Ipecac, 2 grs.—M.

Which was ordered to be given in syrup at bed time, and passed off the next morning with castor oil and turpentine.

The same prescription was repeated for four consecutive nights, at the expiration of which time the patient was cured.

In 1856, two of Mr. F's grown sons were taken with dysentery about the same time. I treated them with sulphate magnesia and small doses sulphate morphine, for eight days.

By this treatment one of them was relieved; but the other evidently grew worse, to whom I then gave calomel, grs. xx, opium, gr. 1, ipecac, grs. 11, which was repeated for two consecutive nights. The patient convalesced rapidly, and further treatment was not called for.

I will give one more illustration on a large scale, founded on my observations during the war. I was ordered on duty, with the 17th Kentucky Regiment, one month before the battle of Shilo, at which place I met with the Regiment.

Upon inquiry, I found seventy of the men disabled from duty, chiefly from camp diarrhœa. Beside, I learned that a large number had died, been discharged from the service, and sent to general hospitals. The treatment had been small doses of calomel, blue mass, opium, and other astringents. I changed the prescription, and gave scruple doses of calomel to all the fresh cases as they occurred, which relieved them in a few days, and thus prevented them from running into the chronic form.

The chronic cases on hand, when I joined the regiment, that did not recover during the spring, were cured by green corn in the summer, without any further medication. The green corn was scraped from the cob and boiled done in water, a piece of fat pork being thrown in to season it. Thus the regiment was delivered from that terrible army scourge.

In the fall of the same year, I took charge, as Surgeon, of the 35th Regiment of Kentucky Mounted Infantry—a new Regiment just brought into the service. I treated all the cases of camp diarrhœa as they occurred, with large doses of calomel. I served with the regiment six months, and can now truthfully say that not a man died, not one was discharged from service, or sent to general hospital on account of diarrhœa, during that time, as my monthly reports will show.

After I left the regiment, my assistant surgeon continued the same treatment; and as it was connected with General Hobson's command, on whose staff I was serving, I had the opportunity to know that the prescription succeeded until the regiment was mustered out, just prior to the close of the war.

I do not always administer calomel for the relief of dysentery and diarrhœa. A large majority of the cases in this locality will yield to a milder and more popular plan of treatment. But when this plan does fail, and I deem it necessary to give calomel, I give it in large doses, especially in all fresh and acute cases.

But I will further support the correctness of my position by the authority of others, who have enjoyed the most favorable opportunities of arriving at truthful results.

Dr. Armesly, who practiced many years in India, says that "calomel combines with, and renders fluid, and detaches the viscid mucous secretions attached to the alimentary canal; it diminishes the vascular state of the stomach when it is in excess, and increases the capillary circulation in the mucous coat of the large intestines.

Hence, it is useful in large doses in increased vascular action of the intestinal canal indicated by the state of the tongue and irritability of the stomach, such as occurs in fever, hepatitis, dysentery and peritonal inflammation after full bleeding." He further remarks in another place: "It is generally believed, and probably may be true, that many constitutions in India are ruined by the use of calomel; but I am disposed to consider this to be the consequence of continuing it in small quantities long after the necessity for using it ceases to exist. Small doses of calomel, from two, three to four grains, will purge and keep up a considerable degree of irritation in the stomach and bowels, when twenty grains will not; but on the contrary, will allay the irritation of both when it results from the inflammation of their mucous surfaces."

Dr. Johnson says on the same subject: "I shall prove in the course of this essay, what indeed is well known to many of my brother officers who have served in India, that twenty grains of calomel will act as a sedative, and so far from griping and producing hypocatharsis, will soothe uneasiness, and rather constipate than purge."

By Dr. Merrill, of Machez, it is said: "Calomel, when given alone, I have always found to produce the best effect in scruple doses. A smaller quantity than this operates more frequently, producing greater irritation of the stomach and bowels, and causing frequent watery dejections, which rapidly debilitate the patient."

Again he says: "The medium dose of twenty grains rarely fails to quiet the irritability of the stomach and bowels and carry off large quantities of feculent bilious matter without griping, tenesmus, prostration, or any other untoward symptom."

Dr. Cartwright, in his essay on syphilis, remarks that "those who have not used calomel extensively, would be apt to suppose a *priori* that large doses would produce hypocatharsis, and debilitate the patient; but experience can best refute such suppositions, for it shows us that large doses of calomel operate much more mildly than small ones."

Dr. Armstrong says: "Small doses of calomel, from two to four and six grains, will purge and keep up a considerable degree of irritation in the stomach and bowels, when twenty grains will not, but on the contrary, will allay the irritation of both, when it results from inflammation of their mucous surfaces."

Dr. Thompson, in his work on Materia Medica and Therapeutics, informs us, that "it often happens that small doses of calomel cannot be retained on the stomach when this viscous is in an irritable state, although it retains large doses, which act as a sedative."

Now it does seem to me, that the concurrent testimony of so many respectable physicians, who have formed their opinions from actual observations, ought to satisfy all that the effects of a large dose of calomel are very different from those of a small one; and that while the former allays the irritation of the bowels, the latter rather augments it than otherwise.

